# Estimates of vaccine effectiveness against measles and mumps: 14 years follow-up of a large cohort in Wales, UK

**DOI:** 10.1093/ije/dyag083

**Published:** 2026-06-06

**Authors:** Malorie Perry, Michael B Gravenor, Simon Cottrell, Catherine Moore, Lucy J Griffiths

**Affiliations:** Population Data Science, Health Data Research UK, Swansea University Medical School, Swansea, SA2 8PP, Wales, United Kingdom; Vaccine Preventable Disease Programme and Communicable Disease Surveillance Centre, Public Health Wales, Cardiff, CF10 4BZ, Wales, United Kingdom; Population Data Science, Health Data Research UK, Swansea University Medical School, Swansea, SA2 8PP, Wales, United Kingdom; Vaccine Preventable Disease Programme and Communicable Disease Surveillance Centre, Public Health Wales, Cardiff, CF10 4BZ, Wales, United Kingdom; Wales Specialist Virology Centre, University Hospital of Wales, Cardiff, CF14 4XW, Wales, United Kingdom; Population Data Science, Health Data Research UK, Swansea University Medical School, Swansea, SA2 8PP, Wales, United Kingdom

**Keywords:** vaccination, immunization, effectiveness, measles, MMR, measles–mumps–rubella vaccine

## Abstract

**Background:**

Uptake of the measles–mumps–rubella vaccine in Wales is high. However, sporadic measles cases still occur and there are large mumps outbreaks every few years. In this study, the long-term vaccine effectiveness (VE) of vaccines containing measles and mumps is assessed.

**Methods:**

A retrospective cohort of 822 116 individuals aged 1–30 years were followed up between 1 January 2007 and 31 December 2020. Welsh Demographic Service data were linked to vaccination status from the national vaccination register and primary care records. Outcomes were identified by linking to laboratory confirmations (measles and mumps) and notifications (mumps) data. Complications were sourced from hospital admissions and primary care data. Extended Cox regression was used to calculate hazard ratios.

**Results:**

The adjusted VE (aVE) against confirmed measles after two doses remained high after 15 years 99.7% [95% confidence interval (CI) 99.2–99.9]. The aVE for confirmed mumps was lower, with decline over time: 93.6% (95% CI 90.2–95.8) in the first 5 years after vaccination with dose two and 49.9% (95% CI 34.4–61.8) after ≥15 years. A third dose of mumps vaccine temporarily increases protection (87.6%, 95% CI 71.7–94.6). The aVE estimates for mumps were lower when based on clinical suspicion. The VE was high against complications for both infections.

**Conclusion:**

The high, sustained VE for measles strengthens evidence that elimination remains possible and the high VE against mumps complications is encouraging. Evidence for the waning of mumps immunity may be important when deciding to implement a third dose in outbreak settings. With the increased use of data linkage, studies should be conducted to corroborate these findings.

Key Messages

- The aim of this study was to assess the long-term vaccine effectiveness (VE) of vaccines containing measles and mumps against confirmed infection and complications by using a large cohort in Wales, UK.
- The VE 15 years after two doses of measles vaccine remained at >95%, whilst the effectiveness of the mumps vaccine began to decline after 5 years, although the effectiveness against complications remained high.
- The high, sustained VE for measles strengthens evidence that elimination remains possible whilst the waning effectiveness for mumps suggests that a third dose in outbreaks may be beneficial.

## Introduction

Vaccination against measles has been available in Wales since 1968 [1]. However, uptake of the single-antigen vaccine was low and measles remained common until the introduction of the measles–mumps–rubella (MMR) vaccine in 1988. A two-dose schedule has been in place since 1996, with doses given at 12–13 months and 3 years 4 months [2]. An additional dose can be given prior to 12 months for protection during outbreaks [1]. Vaccinations are free of charge through the National Health Service (NHS). Uptake ranged from 78% to 98% for one dose (at 2 years) and 71% to 94% for two doses (at 5 years) between 2000 and 2020 [3]. High coverage is key to the WHO European Region measles and rubella elimination goal [4]. Vaccinations for residents ≤18 years of age are recorded in a national register—the Children and Young Persons Integrated System—in addition to primary care general practitioner (GP) records.

The MMR vaccine has been highly effective against rubella, with no confirmed cases in Wales since 2005. However, sporadic measles cases occur, with the last large outbreak in 2013 (>1000 cases) [5]. Large outbreaks of mumps occur every few years [6]. Suspected cases of measles and mumps are legally notifiable and recorded in the national case-management system. Following local guidelines, all suspected measles cases should be sent two test kits. An oral fluid sample is returned to the UK Health Security Agency (UKHSA) reference laboratory for serology testing and one sample is sent for polymerase chain reaction (PCR) testing at the Wales Specialist Virology Centre, for rapid confirmation. Oral fluid testing for mumps is also performed by UKHSA, although the proportion of suspected mumps cases tested is low, especially during times of high incidence. Notifications are often used as an indicator of mumps activity.

Although the vaccine effectiveness (VE) of MMR against measles has been shown to be >95%, most estimates are based on attack rates in outbreak settings [7–10]. Larger studies are based on clinical diagnosis rather than confirmations, which leads to underestimates of effectiveness [11–13]. The VE of MMR against mumps infection is known to be lower than that against measles, with evidence of waning [14–17]. However, there are no large studies of the VE of two doses against confirmed infection. Studies with small sample sizes are often not adequately powered for precise results and detailed evidence of waning is unavailable [18, 19]. Large cohort studies are important in providing robust estimates for monitoring the ongoing effectiveness of vaccinations and country-specific data may help to increase local vaccine confidence.

The aim here was to assess the long-term VE of vaccines containing measles and mumps against (i) confirmed measles infection and (ii) notified/confirmed mumps infection. As a secondary aim, the VE against complications was assessed.

## Methods

Analyses were completed within the Secure Anonymised Information Linkage (SAIL) Databank—a repository of anonymized individual-level national datasets [20]. A retrospective cohort study of individuals aged 1–30 years on 31 December 2020 (born in 1990–2019) and alive and resident in Wales between 1 January 2007 and 31 December 2020 was constructed. A total of 1 327 297 eligible individuals were identified by using the Welsh Demographic Service Dataset—a national demographic register of those registered for NHS care. Vaccination data were obtained from the National Community Child Health Database (NCCHD), extracted from the Children and Young Persons Integrated System and supplemented with data from primary care GPs (WLGP), as described previously [21].

For exclusions, see Supplementary Material Section 1. Confirmations for measles were obtained from reconciled UKHSA and Wales Virology Centre test data that are used for routine national surveillance. Notifications for mumps were linked to UKHSA serology results. Cases that tested negative were removed from the notified case list and treated in the same way as non-cases. Confirmed, equivocal, or untested (notified only) cases were imported into SAIL. When the onset date was unavailable, the earliest of the notification date or sample-collection date was used.

Complications were sourced from hospital admissions data (Patient Episode Dataset for Wales) and WLGP (Supplementary Material Section 2). Complications were included if the hospital admission date or GP consultation date occurred 7 days before or 28 days after the reported onset date. Individuals entered the study on the latest of: 1 January 2007, their first birthday, or the date on which they first moved into or registered with a GP in Wales. Individuals were censored at the earliest of: the onset date, the date they moved out of Wales, the date of death, or 31 December 2020 (Supplementary Figure 1).

An extended Cox regression model, as described by Zhang *et al*. [22], was used to calculate hazard ratios (HRs), where VE = 1 – HR = 1-exp(γ)


$$h(t)=h_{0}(t)\;\times \exp(b_{1}x_{1}+\;b_{2}x_{2}\ldots \;b_{n}x_{n}+\;\gamma Xg(t))$$

Vaccination was introduced as a time-varying covariate [Xg(t)] i.e. individuals vaccinated with their first dose after study entry contributed an unvaccinated amount of person-time, before contributing person-time to the one-dose vaccination category. On receipt of a second dose, they would end their time contributed to the one-dose category and begin to contribute time to the two-dose vaccination category, and so on. The vaccination dates were adjusted by 14 days to account for the time needed for immunity to develop. Adjusted analysis included categorical covariates ($x_{n}$) that were significant at the 0.2 level in univariable analysis (Supplementary Table 1), the derivation of which has been described previously [21].

The baseline for estimates was the unvaccinated population. Individuals with confirmed (and notified, for mumps) infection but no recorded complication were excluded from estimates against complications. Data for missing adjusting variables [birth order (18.3%), broad ethnic group (8.4%), free school meals (4.4%)] were imputed using Multivariate Imputation by Chained Equations and the Nelson–Aalen estimator [23, 24]. Twenty iterations were performed using all model variables including the outcome, with no auxiliary variables [25]. Polytomous logistic regression was used for birth order and free school meals, and logistic regression for ethnic group.

Analyses were carried out by using R version 4.1.3.

## Results

The final cohort included 822 116 individuals (Supplementary Table 1). Of these, 96.8%, 85.5%, and 6.1% received one, two, and three doses of a measles-containing vaccine, with a lower coverage for mumps (96.8%, 84.8%, and 4.9%, respectively). A small proportion (<3%) had single-antigen vaccines. The vaccination rate within 6 months of the due date was 93% for the first dose and 63% for the second dose.

From the population in the final cohort, there were 568 confirmed measles cases, with 88 complications [15.5%, 95% confidence interval (CI) 12.7–18.7]. The age at onset ranged from 1 to 25 years (Supplementary Table 6). The adjusted vaccine effectiveness (aVE) against confirmed infection was 94.7% (95% CI 93.1–96.0) for one dose, increasing to 99.5% (95% CI 99.4–99.7) for two doses, with similar estimates after three doses [99.6% (95% CI 98.7–99.9)] (Table 1). Against any complication, the aVE was 94.3% (95% CI 88.9–97.1), increasing to 99.7% (95% CI 99.2–99.9) for two doses (Table 1).

**Table 1:** HRs comparing vaccinated with unvaccinated individuals for confirmed measles infection and for complications in confirmed cases in a retrospective cohort of 822 116 people aged 1–30 years in Wales, UK.^a^

Outcome	Category	Individuals	Rate per 100 000 person-days^b^	Unadjusted HR (95% CI)			Adjusted HR (95% CI)		
Confirmed	Unvaccinated	399 410	0.209	–			–		
measles infection	One dose	475 027	0.015	0.038 (0.029–0.050)			0.053 (0.040–0.069)		
	Two doses	675 008	0.002	0.004 (0.003–0.005)			0.005 (0.003–0.006)		
	Three doses	49 851	0.002	0.004 (0.001–0.011)			0.004 (0.001–0.013)		
Complications	Unvaccinated	389 930	0.034	–			–		
with confirmed	One dose	462 680	0.003	0.047 (0.025–0.091)			0.057 (0.029–0.111)		
measles infection	Two doses	652 658	<0.001	0.002 (0.001–0.007)			0.003 (0.001–0.008)		
	Three doses^c^	N/a	N/a	N/a			N/a		

a HRs calculated by using extended Cox regression models. Adjusted estimates adjusted for: age as at 31 December 2020, gender, birth order, age first registered with a Wales GP, broad ethnic group, Health Board of residence, deprivation quintile of residence, eligibility for free school meals, total GP visits in the year preceding 31 December 2020, and residence in a rural/urban area. VE is calculated as 1 – HR. Dash indicates data not applicable.

b Rate per 100 000 person-days calculated by using the number of outcome events occurring in each vaccination category, divided by the total number of person-days, multiplied by 100 000. Individuals can contribute person-time to multiple vaccination categories as their vaccination status changes over time.

c Estimates for VE of three doses of measles-containing vaccine against complications with confirmed infection unavailable due to small numbers.

**Figure 1:**
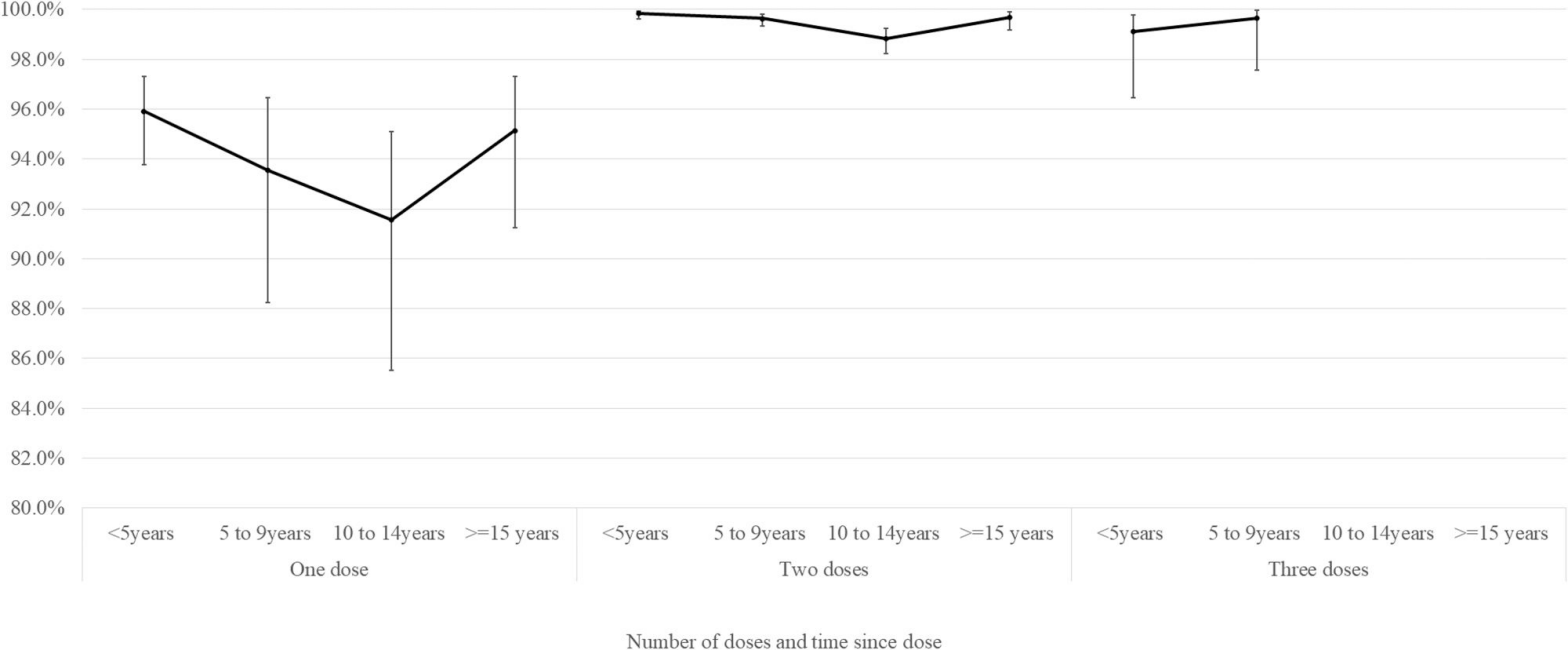
Estimated VE for measles-containing vaccine against laboratory-confirmed measles infection by time since dose in a retrospective cohort of 822 116 people aged 1–30 years in Wales, UK.^a^ ^a^Cox regression model adjusted for age as at 31 December 2020, gender, birth order, age first registered with a Wales GP, broad ethnic group, Health Board of residence, deprivation quintile of residence, eligibility for free school meals, total GP visits in the year preceding 31 December 2020 (or year before they exited the study), and residence in a rural/urban area. Underlying data and parameter estimates for adjustment variables can be found in Supplementary Table 10.

Over time, estimates for the effectiveness of one dose of measles vaccine against confirmed infection decreased slightly, although the CIs overlapped at all time points (Fig. 1). There was no evidence of waning following two doses, with the aVE in the first 5 years since vaccination at 99.8% (95% CI 99.6–99.9) and 99.7% (95% CI 99.2–99.9) for ≥15 years since vaccination. There were too few outcomes to estimate the waning after three doses.

There were 1163 confirmed mumps cases, with an additional 2340 notified but not tested, and 42 complications. The age at onset ranged from 1 to 30 years (Supplementary Table 8). The aVE for one dose of mumps-containing vaccine against suspected or confirmed infection was 50.5% (95% CI 41.5–58.0), with estimates against two doses at 63.5% (95% CI 58.0–68.3) and those against three doses at 71.8% (95% CI 65.0–77.2). With only confirmed cases included, the aVE increased to 71.5% (95% CI 61.9–78.7), 76.0% (95% CI 70.1–80.7), and 84.5% (95% CI 77.8–89.2), respectively (Table 2). The aVE against complications was higher, at 80.6% (95% CI 40.2–93.7) for one dose, increasing to 94.4% (95% CI 86.4–97.7) for two doses and 93.5% (95% CI 62.0–98.9) for three doses (Table 2).

**Table 2:** HRs comparing vaccinated with unvaccinated individuals for clinically notified mumps infection, confirmed infection, and complications in confirmed cases in a retrospective cohort of 822 116 people aged 1–30 years in Wales, UK.^a^

Outcome	Category	Individuals	Rate per 100 000 person-days^b^	Unadjusted HR (95% CI)			Adjusted HR (95% CI)		
Suspected	Unvaccinated	400 263	0.115	–			–		
and/or confirmed	One dose	479 658	0.094	0.484 (0.409–0.573)			0.495 (0.420–0.585)		
mumps infection	Two doses	677 764	0.122	0.402 (0.348–0.464)			0.365 (0.317–0.420)		
	Three doses	40 571	0.108	0.332 (0.267–0.411)			0.282 (0.228–0.350)		
Confirmed	Unvaccinated	399 452	0.043	–			–		
mumps infection	One dose	478 673	0.020	0.271 (0.200–0.369)			0.285 (0.213–0.381)		
	Two doses	675 961	0.043	0.314 (0.248–0.397)			0.240 (0.193–0.299)		
	Three doses	40 472	0.034	0.230 (0.159–0.333)			0.155 (0.108–0.222)		
Complications	Unvaccinated	399 290	0.005	–			–		
with confirmed	One dose	478 466	0.002	0.170 (0.062–0.468)			0.194 (0.063–0.598)		
mumps infection	Two doses	675 040	0.001	0.057 (0.026–0.124)			0.056 (0.023–0.136)		
	Three doses	40 429	0.002	0.078 (0.017–0.362)			0.065 (0.011–0.380)		

a HRs calculated by using extended Cox regression models. Adjusted estimates adjusted for: age as at 31 December 2020, gender, birth order, age first registered with a Wales GP, broad ethnic group, Health Board of residence, deprivation quintile of residence, eligibility for free school meals, total GP visits in the year preceding 31 December 2020, and residence in a rural/urban area. VE is calculated as 1 – HR. Dash indicates data not applicable.

b Rate per 100 000 person-days calculated by using the number of outcome events occurring in each vaccination category, divided by the total number of person-days, multiplied by 100 000. Individuals can contribute person-time to multiple vaccination categories as their vaccination status changes over time.

**Figure 2:**
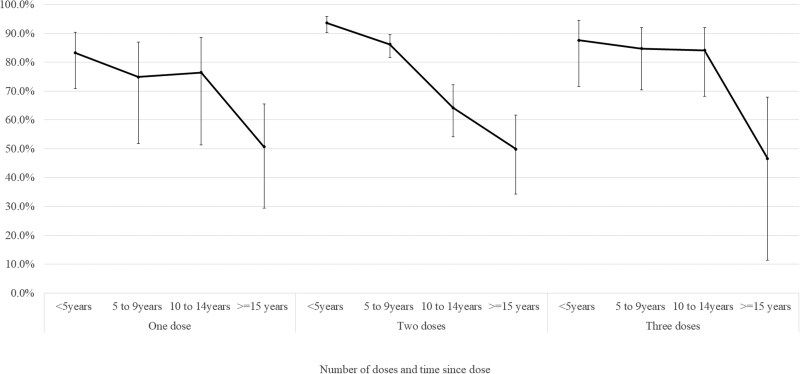
Estimated VE for mumps-containing vaccine against laboratory-confirmed mumps infection by time since dose in a retrospective cohort of 822 116 people aged 1–30 years in Wales, UK.^a^ ^a^Cox regression model adjusted for age as at 31 December 2020, gender, birth order, age first registered with a Wales GP, broad ethnic group, Health Board of residence, deprivation quintile of residence, eligibility for free school meals, total GP visits in the year preceding 31 December 2020 (or year before they exited the study), and residence in a rural/urban area. Underlying data and parameter estimates for adjustment variables can be found in Supplementary Table 11.

The aVE against confirmed infection decreased with time since the second dose of mumps-containing vaccine (Fig. 2). In the first 5 years since vaccination with a second dose, the aVE was estimated to be 93.6% (95% CI 90.2–95.8), declining to 86.3% (95% CI 81.7–89.7) at 5–9 years and 49.9% (95% CI 34.4–61.8) after 15 years. Increases in the aVE were seen after a third dose [87.6% (95% CI 71.7–94.6)], with the aVE estimates remaining similar over time.

## Discussion

This large study shows high VE against measles, with no strong evidence of waning 15 years after receiving a complete course. The VE for mumps-containing vaccines was lower, with a decline 4 years after dose two, although a third dose appears to increase protection for several years. The VE was lower when based on clinical suspicion compared with confirmed cases only, as expected due to possible case misclassification. The VE was high against complications for both infections. For those with confirmed measles, the complication rates were high (>15%).

Comparing estimates from other studies is challenging, as vaccination schedules vary, different vaccines may be in use, and delivery settings can impact effectiveness. Underlying population immunity due to natural infection can also affect estimates. Many VE estimates are derived from small cohorts in outbreak settings by using attack rates as described by Orenstein *et al.* or the screening method as described by Farrington [26, 27]. Although these are convenient methods, estimates can vary and be imprecise. In line with the estimates found here, a review of 70 published studies showed a median VE of 92.0% (interquartile range 86%–96%) for one dose of measles-containing vaccine given at ≥12 months of age [7]. Estimates for the two-dose VE against confirmed infection have been as low as 63.4% (95% CI –103.0 to 90.6) in some settings in which primary vaccine failure due to poor handling has been cited [28]. However, estimates of >99% are not uncommon [29–31]. The one-dose VE in a previous Wales outbreak was estimated to be 99.3% (95% CI 90–100) [32].

The Joint Committee on Vaccination and Immunisation has advised that the second dose of MMR vaccine in the UK should be moved to 18 months of age to improve coverage [33]. This schedule has been shown to confer good protection in other countries, with estimates of 99.7% (95% CI 99.2–99.9) for two doses against confirmed measles infection in an Australian study and slightly lower estimates from Canada [10, 34].

Waning protection from the measles vaccine was not observed in this cohort. The maximum age of individuals in this cohort was 30 years, with the oldest case 25 years. Therefore, the identification of longer-term waning will need longer follow-up. Previous studies in France and the UK using clinically diagnosed cases observed slight waning, but estimates were imprecise [35, 36]. Other studies in which VE was estimated by using Cox regression considered one dose of measles-containing vaccine only, but gave similar results against a confirmed-case outcome [11, 12, 37]. The antibody concentration in vaccinated individuals is known to decline but is not observed following natural infection [38, 39]. Those born prior to 1990 would have been more likely to have experienced natural measles infection and therefore have lifelong immunity [2]. Identifying these individuals is challenging, with a lack of historical electronic records. Recent modeling suggests that vaccination is highly protective for several decades, although there may be increases in breakthrough infections after 15 years [40].

Vaccines containing the mumps Jeryl Lynn strain (which has been the exclusive strain used in the UK since the end of 1992) demonstrated high efficacy under clinical trial conditions, but a lower VE in outbreak settings [14]. Many studies use clinical diagnosis rather than confirmed infection, which may underestimate the VE, as this study suggests. Estimates using clinical diagnosis report the VE for one dose of vaccine to be from 30% to >90% [9, 14, 15, 41–45]. The VE against clinically diagnosed infection for two doses has been as high as 93% (95% CI 85–97) [41] but has been commonly reported at 60%–80% [15, 42, 44–46].

In the absence of PCR testing, confirming mumps infection in previously vaccinated individuals is challenging, which may lead to overestimates of VE [47]. Estimates from studies using confirmed cases report VE as high as 94.6% (95% CI 92.9–95.9) for two doses [16, 19, 48]. In contrast, Harling *et al.* compared clinical diagnosis with confirmed infection as the outcome, finding similar estimates for at least one dose [17]. Using a more specific clinical case definition results in increased VE [49]. Estimates here, using suspected or confirmed cases, appear to be low compared with those of other studies using clinically diagnosed cases. It is possible that, in outbreak settings, the positive predictive value is higher and the proportion of suspected cases that are true infections is higher, resulting in more accurate VE. Higher exposure in household studies may result in lower VE [41].

Orlíková *et al.* estimated the VE of two doses of mumps vaccination against any complication at 68% (95% CI 61–75), similar to the 76% (95% CI 61–86) reported by Sane *et al.* and 62.7% (95% CI 25.7–81.3) by Zamir *et al*. [50–52]. For orchitis, Hahné *et al.* reported a VE of 74% (95% CI 49–87) [53]. However, these studies consider individuals with mumps and estimate the VE against developing a complication. Therefore, here, the slightly higher estimate of 94.4% (95% CI 86.4–97.7) for two doses against mumps complications, where the comparison group are individuals who did not acquire any mumps infection, may have been expected.

It has been estimated that the immunity derived from mumps vaccination wanes after ∼27 years (95% CI 16–51) [15], with the likelihood of complications increasing with time since the second dose [50]. However, in some outbreaks, notable decreases in VE over time since vaccination have not been seen [15]. It was not possible to precisely measure waning following one dose of mumps vaccine, as people typically have a second dose within 5 years, reducing the person-time after for analysis. Findings here may support the use of a third dose in some outbreak settings, although, unfortunately, it was not possible to assess whether the effectiveness against complications declines over time.

This study has a number of limitations. Although a national vaccination register was used, misclassification of the vaccination status may have existed. However, there is no reason why this should differ between cases and non-cases. This study only includes people registered with a primary care GP in Wales. Excluding these individuals aims to reduce bias due to uncertain vaccination status. However, if those who are unregistered have not consulted for infection and are less likely to be vaccinated, then this would underestimate the VE. Those who moved to Wales after 18 years of age, from elsewhere in the UK, will not be present in the NCCHD and were therefore excluded. This led to a larger proportion of older individuals excluded from the study, although this was necessary in order to reduce bias due to uncertain vaccination status. Additionally, only individuals registered with practices that submit data to SAIL are included, although these practices are representative of the total population registered for NHS care [54].

It was important to ensure equal exposure between vaccinated and unvaccinated groups. The recorded MMR vaccination coverage in Wales is lower in those born outside of the UK [21]. Previous exposure and infection are also more uncertain for these individuals. If natural immunity is higher and vaccination coverage lower, then this will underestimate the VE. Therefore, the population was restricted to UK-born individuals, so that the lifetime exposure would be similar for this age group. However, within the UK, there are areas that have a higher prevalence. It was not possible to exclude individuals who had had measles/mumps infection prior to 2007 due to a lack of historical records or to identify those who had had an infection before they moved to Wales. Individuals may have been infected but did not seek health advice or get tested. The under-ascertainment of cases will bias towards the null.

It was also important that case ascertainment was equal between the exposure groups. Vaccinated cases may have had a milder attenuated infection that could have been under-diagnosed, leading to overestimates of the VE [55–57]. A number of complications from infection were considered, although rarer complications such as oophoritis (from mumps) and deafness (from measles) were not. This is not expected to have impacted the overall estimates. General practitioners’ ability to recognize and associate complications with previous infection may be limited in a country where transmission is not endemic.

A strength of this study is the ability to adjust for a number of factors. Missing data for a small number of independent variables were imputed to retain power. Data were assumed to be missing at random, which may not hold true. More suitable methods for imputation may exist. Analysis using a complete-case approach showed similar estimates for VE (data not presented).

Different VE estimates may have been observed in populations excluded from this analysis. For example, the overall rate of measles infection was 38.9 per 100 000 population in the excluded non-UK-born individuals over the study period compared with 73.8 per 100 000 in the UK-born study population. For mumps, this was higher, at 198.6 in the non-UK-born and 426.2 in the UK-born. Vaccination coverage is lower in the non-UK-born population, but case ascertainment may also differ, particularly if infection is seen as common in childhood. Individuals may have had infection as a child and vaccinations may also have been given in less optimal settings, which can reduce the VE. Individuals vaccinated before 12 months of age were excluded, as maternal antibodies may interfere with seroconversion following vaccination. It is common for individuals to be vaccinated earlier in settings in which the risk of infection is high. Individuals receiving an alternative schedule are likely to have some long-term protection [58]. There is need to continuously review the timing of measles vaccine doses in the context of elimination, as schedules may need to be altered in areas where there is high susceptibility before the first dose is due [59].

## Conclusions

This is the largest known VE study for measles and mumps infection and associated complications. Precise estimates over time were calculated by using a national vaccination registry and confirmed infection as the outcome. The high, sustained VE seen for measles strengthens evidence that elimination remains possible. Longer periods of follow-up are required to estimate the VE in older individuals, 15 years after vaccination.

The high VE for mumps against complications is encouraging. The evidence for waning immunity, even 5 years after dose two, may be useful when deciding to implement a third dose in outbreak settings. The need to fully understand the waning protection against mumps over time is important in understanding and planning for potential implications of changes in vaccination schedules. Although the majority of individuals received vaccination at around the time it was due, further studies would be needed to assess the impact of late (or early) vaccination.

This study highlights the importance of accurate classification of cases in VE studies and also the challenges with interpreting estimates from outbreak settings. Depending on the age of the population, different levels of waning immunity are likely to be present, which makes results difficult to interpret. With the increasing availability of data linkage, the use of survival analysis and modeling should allow untangling of the issues of age distribution and waning immunity, such as has been demonstrated in this paper.

## Ethics approval

All analyses were performed within the SAIL Databank—a repository of anonymized individual-level national datasets, hosted by Swansea University. All proposals to use SAIL data are subject to review by an independent Information Governance Review Panel (IGRP). Before any data can be accessed, approval must be given by the IGRP. The IGRP gives careful consideration to each project to ensure the proper and appropriate use of SAIL data. This project was undertaken as part of IGRP-approved project 0899. Notifications and confirmations data for the cohort study period were imported into SAIL and anonymized for the purpose of this study, following local Data Protection Impact Assessment procedures.

## Supplementary Material

dyag083_Supplementary_Data

## Data Availability

The data used in this study are available from the SAIL Databank at Swansea University, Swansea, UK, which is part of the national e-health records research infrastructure for Wales. All proposals to use SAIL data are subject to review by an independent IGRP. Before any data can be accessed, approval must be given by the IGRP. The IGRP gives careful consideration to each project to ensure the proper and appropriate use of SAIL data. When access has been approved, it is gained through a privacy-protecting safe haven and remote-access system referred to as the SAIL Gateway. SAIL has established an application process to be followed by anyone who would like to access data via SAIL at www.saildatabank.com/application-process. Model code has been made available online at https://doi.org/10.5281/zenodo.17716573.
