# Supplementary material for: Estimates of vaccine effectiveness against measles and mumps: 14 years follow-up of a large cohort in Wales, UK
Source: Int J Epidemiol. 2026 Jun 6;55(3):dyag083. doi: 10.1093/ije/dyag083 (PMC13242217; doi:10.1093/ije/dyag083)
Supplement: dyag083_Supplementary_Data [file dyag083_supplementary_data.docx]

**Supplementary Material**

**Contents Page**

**Section 1** Description of exclusions applied to the study population and a summary of the cohort included in analysis of measles and mumps vaccine effectiveness, Wales UK…………………….… 4

**Supplementary Table S1.** Descriptive summary of 822 116 one- to 30-year-olds in a retrospective cohort study of measles and mumps vaccine effectiveness, Wales UK………………………………. 4

**Section 2.** Code lists for identify complications in measles and mumps cases………………………. 6

**Supplementary Table S2.** ICD-10 codes used to identify measles related hospital admissions in the Patient Episode Dataset for Wales dataset……………………………………………………………. 6

**Supplementary Table S3.** ICD-10 codes used to identify mumps related hospital admissions in the Patient Episode Dataset for Wales dataset……………………………………………………………. 7

**Supplementary Table S4.** Read v2 codes used to identify additional complications from measles related infections in the Primary Care GP consultations dataset………………………………………7

**Supplementary Table S5.** Read v2 codes used to identify additional complications from mumps

related infections in the Primary Care GP consultations dataset………………………………………8

**Section 3.** Description of cohort follow-up process…………………………………………………...10

**Supplementary Figure S1.** Diagram illustrating various scenarios of follow-up, including inclusion and censoring points. Individuals born between 1990 and 2019 were eligible for inclusion………….10

**Section 4.** Summary of measles and mumps cases in a retrospective cohort study of vaccine effectiveness, Wales UK……………………………………………………………………………….12

**Supplementary Table S6.** Descriptive summary of confirmed measles cases, as at date of onset, in a retrospective cohort study of vaccine effectiveness, Wales UK……………………………………….12

**Supplementary Table S7.** Descriptive summary of confirmed measles cases with complications, as at date of onset, in a retrospective cohort study of vaccine effectiveness, Wales UK……………………13

**Supplementary Table S8.** Descriptive summary of confirmed mumps cases, as at date of onset, in a retrospective cohort study of vaccine effectiveness, Wales UK…………………………………….…14

**Supplementary Table S9.** Descriptive summary of confirmed mumps cases with complications, as at date of onset, in a retrospective cohort study of vaccine effectiveness, Wales UK……………………15

**Section 5.** Vaccine effectiveness estimates for measles and mumps vaccination over time, as shown in Figure 1 and Figure 2……………………………………………………………………………….....16

**Supplementary Table S10.** Estimated vaccine effectiveness for measles containing vaccine against laboratory confirmed measles infection by time since dose, in a retrospective cohort of 822 116 one to 30 year olds in Wales, UK……………………………………………………………………………..16

**Supplementary Table S11.** Estimated vaccine effectiveness for mumps containing vaccine against laboratory confirmed mumps infection by time since dose, in a retrospective cohort of 822 116 one to 30 year olds in Wales, UK………………………………………………………………………….….18

**Section 6.** Sensitivity analysis of Vaccine Effectiveness estimates for measles and mumps vaccination over time. Cox-regression analysis using an underlying calendar time scale…………………………20

**Supplementary Figure S2.** Estimated vaccine effectiveness for measles containing vaccine against laboratory confirmed measles infection by time since dose, in a retrospective cohort of 822 116 one to 30 year olds in Wales, United Kingdom……………………………………………………………….20

**Supplementary Table S12.** Estimated vaccine effectiveness for measles containing vaccine against laboratory confirmed measles infection by time since dose, in a retrospective cohort of 822 116 one to 30 year olds in Wales, United Kingdom……………………………………………………………….21

**Supplementary Figure S3.** Estimated vaccine effectiveness for mumps containing vaccine against laboratory confirmed mumps infection by time since dose, in a retrospective cohort of 822 116 one to 30 year olds in Wales, United Kingdom………………………………………………………………22

**Supplementary Table S13.** Estimated vaccine effectiveness for mumps containing vaccine against laboratory confirmed mumps infection by time since dose, in a retrospective cohort of 822 116 one to 30 year olds in Wales, United Kingdom………………………………………………………………23

**Section 1.** Description of exclusions applied to the study population and a summary of the cohort included in analysis of measles and mumps vaccine effectiveness, Wales UK

The following exclusions were applied to the 1 327 297 eligible individuals identified using the Welsh Demographic Service Dataset; those without a record in the NCCHD dataset (n=185 365, 14.0%), those not registered with a General Practice that submits data to SAIL (n=195 936, 14.8%), those with unknown vaccination dates (n=1210, <0.1%), those with record of more than three measles or mumps containing vaccines (n=9229, 0.7%), those where their first vaccination for measles or mumps was before the recommended age of 12 months (n=19 565, 1.5%) and those who were born overseas, for whom previous measles and mumps exposure is unknown and vaccination history can be less reliable (n=92 658, 7.0%). A further 529 (<0.1%) individuals were excluded because they had a measles or mumps related hospital admission or GP consultation but no notification or confirmation of disease. Additionally, 372 (<0.1%) possible (unconfirmed) measles/mumps cases and 689 (<0.1%) who had confirmed infection before their cohort entry date were excluded.

**Supplementary Table S1.** Descriptive summary of 822 116 one- to 30-year-olds in a retrospective cohort study of measles and mumps vaccine effectiveness, Wales UK. Characteristics listed below are the covariates used in the adjusted vaccine effectiveness estimates.

|  |  | Population | |
| --- | --- | --- | --- |
| Characteristic | Category | (n) | % |
| Age as at 31/12/2020 | 1-4 (2019-2016) | 199189 | 24.2 |
| (Year of birth) | 5-11 (2015-2009) | 101377 | 12.3 |
|  | 12-16 (2008-2004) | 144403 | 17.6 |
|  | 17-18 (2003-2002) | 51618 | 6.3 |
|  | 19-21 (2001-1999) | 76166 | 9.3 |
|  | 22-30 (1998-1990) | 249363 | 30.3 |
| Gender | Male | 421046 | 51.2 |
|  | Female | 401070 | 48.8 |
| Birth order | First born | 300579 | 36.6 |
|  | Second born | 225672 | 27.5 |
|  | Third born | 92874 | 11.3 |
|  | Forth born | 33286 | 4.0 |
|  | Fifth or more | 19190 | 2.3 |
|  | Unknown | 150515 | 18.3 |
| Age first registered with | Born in Wales | 550147 | 66.9 |
| Primary Care GP in Wales | Not born in Wales | 271969 | 33.1 |
| Broad ethnic group | White | 710147 | 86.4 |
|  | Black/Asian/Mixed/Other | 42850 | 5.2 |
|  | Unknown | 69119 | 8.4 |
| Health board of residence | HB1 | 171326 | 20.8 |
|  | HB2 | 108451 | 13.2 |
|  | HB3 | 88202 | 10.7 |
|  | HB4 | 143880 | 17.5 |
|  | HB5 | 138441 | 16.8 |
|  | HB6 | 15452 | 1.9 |
|  | HB7 | 156364 | 19.0 |
| Deprivation quintile | Most deprived | 197933 | 24.1 |
| of residence | 2 | 172475 | 21.0 |
|  | 3 | 152105 | 18.5 |
|  | 4 | 144889 | 17.6 |
|  | Least deprived | 154714 | 18.8 |
| Ever eligible for free | No | 489021 | 59.5 |
| school meals | Yes | 222388 | 27.1 |
|  | Pre-school age | 74358 | 9.0 |
|  | Unknown | 36349 | 4.4 |
| Total Primary Care | 0 | 91966 | 11.2 |
| GP visits 01/01/2020 to | 1-4 | 193847 | 23.6 |
| 31/12/2020 | 5-9 | 172529 | 21.0 |
|  | 10+ | 363774 | 44.2 |
| Rural/Urban area of | Rural | 225863 | 27.5 |
| residence | Urban | 596253 | 72.5 |

**Section 2.** Code lists for identify complications in measles and mumps cases.

The codes listed below were used to identify complications in measles and mumps cases. In a sensitivity analysis, complications were looked for up to 8 weeks post onset. There were <10 additional coded GP complications 4 to 8 weeks post rash onset for both mumps and measles combined. In all of these instances generic codes were used, rather than codes directly associated with measles/mumps. There were no hospital admissions identified 4 to 8 weeks post rash onset. For this reason, 4 weeks (28 days) was used as the cut off for complications included in the VE estimates.

**Supplementary Table S2.** ICD-10 codes used to identify measles related hospital admissions in the Patient Episode Dataset for Wales dataset.

| Code | Description |
| --- | --- |
| B051 | Measles complicated by meningitis |
| A87% | Viral meningitis |
| B050 | Measles complicated by encephalitis |
| G049 | Encephalitis, myelitis and encephalomyelitis, unspecified |
| B052 | Measles complicated by pneumonia |
| J129 | Viral pneumonia, unspecified |
| B053 | Measles complicated by otitis media |
| H671 | Otitis media in viral diseases classified elsewhere |
| H66% | Suppurative and unspecified otitis media |
| B05% (excluding B059) | Measles (excluding Measles without complication) |
|  |  |

**Supplementary Table S3.** ICD-10 codes used to identify mumps related hospital admissions in the Patient Episode Dataset for Wales dataset.

| Code | Description |
| --- | --- |
| B261 | Mumps meningitis |
| A87% | Viral meningitis |
| B262 | Mumps encephalitis |
| G049 | Encephalitis, myelitis and encephalomyelitis, unspecified |
| B263 | Mumps pancreatitis |
| K858 | Other acute pancreatitis |
| K859 | Acute pancreatitis, unspecified |
| B260 | Mumps orchitis |
| N45% | Orchitis and epididymitis |
| B26% (excluding B269) | Mumps (excluding Mumps without complication) |
|  |  |

**Supplementary Table S4.** Read v2 codes used to identify additional complications from measles related infections in the Primary Care GP consultations dataset.

| Code | Description |
| --- | --- |
| A553. | Measles complicated by meningitis |
| F02.. | Meningitis - unspecified cause |
| 65VC. | Notification of acute meningitis |
| F01z. | Meningitis due to organism NOS |
| F011y | Other viral meningitis |
| 1471. | H/O: meningitis |
| A550. | Postmeasles encephalitis |
| F0351 | Encephalitis-post measles |
| F030z | Encephalitis in viral dis NOS |
| 65VB. | Notification of acute encephalitis |
| F035z | Postinfectious enceph NOS |
| F03z. | Encephalitis NOS |
| 1472. | H/O: encephalitis |
| A551. | Postmeasles pneumonia |
| H20z. | Viral pneumonia NOS |
| H2z.. | Pneumonia or influenza NOS |
| A552. | Postmeasles otitis media |
| F52.. | Suppurative otitis media |
| F51.. | Nonsupp otitis media + eustach |
| A55x. | Measles + other spec. complic |
| A55y. | Measles + unspecified complic |
|  |  |

**Supplementary Table S5.** Read v2 codes used to identify additional complications from mumps related infections in the Primary Care GP consultations dataset.

| Code | Description |
| --- | --- |
| A721. | Mumps meningitis |
| F02.. | Meningitis - unspecified cause |
| 65VC. | Notification of acute meningitis |
| F01z. | Meningitis due to organism NOS |
| F011y | Other viral meningitis |
| 1471. | H/O: meningitis |
| A722. | Mumps encephalitis |
| F030z | Encephalitis in viral dis NOS |
| 65VB. | Notification of acute encephal |
| F035z | Postinfectious enceph NOS |
| F03z. | Encephalitis NOS |
| 1472. | H/O: encephalitis |
| A723. | Mumps pancreatitis |
| J6700 | Acute pancreatitis unspecified |
| J670z | Acute pancreatitis NOS |
| A720. | Mumps orchitis |
| K240. | Orchitis |
| A72x. | Mumps+other specif. complicat. |
| A72y. | Mumps with unspecified complic |

**Section 3.** Description of cohort follow-up process.

**Supplementary Figure S1.** Diagram illustrating various scenarios of follow-up, including inclusion and censoring points. Individuals born between 1990 and 2019 were eligible for inclusion.


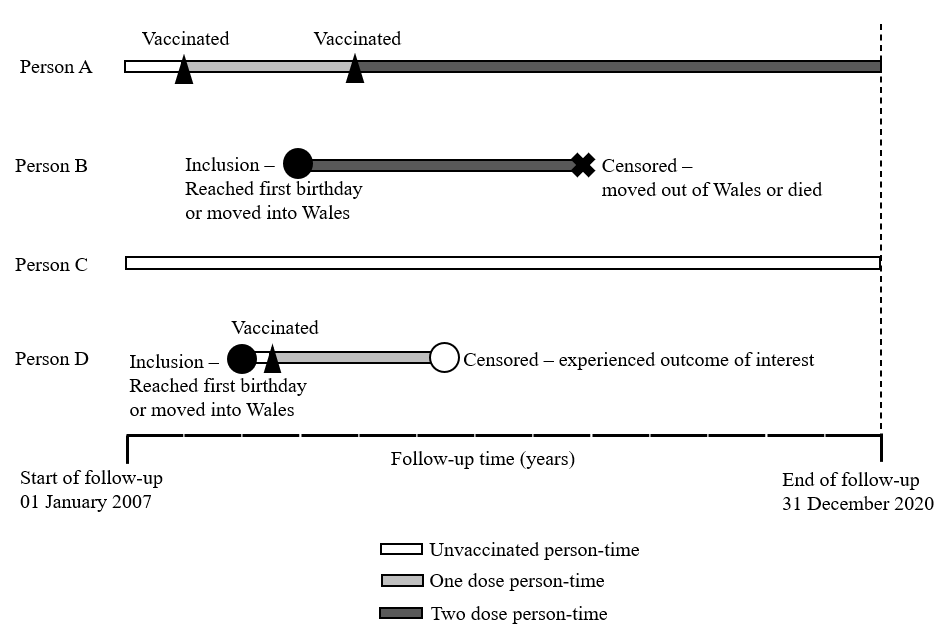


Person A was alive and resident at the start of the follow-up period and were unvaccinated as at 01 January 2007. They received their first dose of MMR after 1 year of follow-up (2008), contributing 1 year of unvaccinated person-time. They received their second dose of MMR 3 years after their first dose (2011), contributing 3 years of one dose person-time. They then remained in the study until the end of the follow-up period 9 years later (2020), contributing 9 years of two dose person-time.

Person B moved into Wales 3 years after the start of follow-up period (2010). They had received two doses of MMR prior to moving into Wales and moved out of Wales after 5 years of being resident (2015). They contributed 5 years of two dose person-time to the study.

Person C was alive and resident at the start of the follow-up period and were unvaccinated as at 01 January 2007. They did not get vaccinated during the 13 year follow-up period and exited the study at the end of the follow-up period (2020) having contributed 13 years of unvaccinated person-time.

Person D entered the study 2 years after the start of follow-up (2009). They were unvaccinated when they entered the study and contributed 6 months of unvaccinated person-time before receiving their first dose of vaccine (mid-2009). They then contributed 3 years of one dose person-time before experiencing the outcome of interest (measles or mumps infection) at which point they were censored (mid-2012).

**Section 4.** Summary of measles and mumps cases in a retrospective cohort study of vaccine effectiveness, Wales UK.

**Supplementary Table S6.** Descriptive summary of confirmed measles cases, as at date of onset, in a retrospective cohort study of vaccine effectiveness, Wales UK. Data have been grouped/suppressed to avoid statistical disclosure.

| Characteristic | Category | (n) |
| --- | --- | --- |
| Age | 1-4 | 132 |
|  | 5-9 | 116 |
|  | 10-14 | 198 |
|  | 15-19 | 100 |
|  | 20-30 | 22 |
| Vaccine doses | 0 | 390 |
|  | 1 | 125 |
|  | 2+ | 53 |
| Health board of residence | HB1 | 44 |
|  | HB2 | 67 |
|  | HB3 | 14 |
|  | HB4 | 33 |
|  | HB5 | 76 |
|  | HB6 | 33 |
|  | HB7 | 301 |
| Deprivation quintile | Most deprived | 142 |
| of residence | 2 | 108 |
|  | 3 | 142 |
|  | 4 | 88 |
|  | Least deprived | 88 |
| Rural/Urban area of | Rural | 133 |
| residence | Urban | 435 |
| Year of onset | 2007 | <10 |
|  | 2008 | <10 |
|  | 2009 | 102 |
|  | 2010 | <10 |
|  | 2011 | 10 |
|  | 2012 | 74 |
|  | 2013 | 316 |
|  | 2014 | <10 |
|  | 2015 | 0 |
|  | 2016 | 11 |
|  | 2017 | 13 |
|  | 2018 | <10 |
|  | 2019 | <10 |
|  | 2020 | 0 |

**Supplementary Table S7.** Descriptive summary of confirmed measles cases with complications, as at date of onset, in a retrospective cohort study of vaccine effectiveness, Wales UK. Data have been grouped/suppressed to avoid statistical disclosure.

| Characteristic | Category | (n) |
| --- | --- | --- |
| Age | 1-4 | 33 |
|  | 5-9 | 11 |
|  | 10-14 | 31 |
|  | 15+ | 13 |
| Vaccine doses | 0 | 55 |
|  | 1+ | 33 |

**Supplementary Table S8.** Descriptive summary of confirmed mumps cases, as at date of onset, in a retrospective cohort study of vaccine effectiveness, Wales UK. Data have been grouped/suppressed to avoid statistical disclosure.

| Characteristic | Category | (n) |
| --- | --- | --- |
| Age | 1-4 | 21 |
|  | 5-9 | 36 |
|  | 10-14 | 119 |
|  | 15-19 | 520 |
|  | 20-24 | 387 |
|  | 25-30 | 80 |
| Vaccine doses | 0 | 94 |
|  | 1 | 92 |
|  | 2 | 932 |
|  | 3 | 45 |
| Health board of residence | HB1 | 171 |
|  | HB2 | 163 |
|  | HB3 | 240 |
|  | HB4 | 199 |
|  | HB5 | 155 |
|  | HB6 | 18 |
|  | HB7 | 217 |
| Deprivation quintile | Most deprived | 220 |
| of residence | 2 | 204 |
|  | 3 | 238 |
|  | 4 | 205 |
|  | Least deprived | 296 |
| Rural/Urban area of | Rural | 331 |
| residence | Urban | 832 |
| Year of onset | 2007 | <10 |
|  | 2008 | <10 |
|  | 2009 | 82 |
|  | 2010 | <10 |
|  | 2011 | 21 |
|  | 2012 | 18 |
|  | 2013 | 162 |
|  | 2014 | 174 |
|  | 2015 | 34 |
|  | 2016 | 17 |
|  | 2017 | 10 |
|  | 2018 | 16 |
|  | 2019 | 307 |
|  | 2020 | 306 |

**Supplementary Table S9.** Descriptive summary of confirmed mumps cases with complications, as at date of onset, in a retrospective cohort study of vaccine effectiveness, Wales UK. Data have been grouped/suppressed to avoid statistical disclosure.

| Characteristic | Category | (n) |
| --- | --- | --- |
| Age | 1-4 | 0 |
|  | 5-19 | 26 |
|  | 20+ | 16 |
| Vaccine doses | 0 | 10 |
|  | 1+ | 32 |

**Section 5.** Vaccine effectiveness estimates for measles and mumps vaccination over time, as shown in Figure 1 and Figure 2. Tables below include parameter estimates for adjustment variables used in the Cox regression models.

**Supplementary Table S10.** Estimated vaccine effectiveness for measles containing vaccine against laboratory confirmed measles infection by time since dose, in a retrospective cohort of 822 116 one to 30 year olds in Wales, UK. Vaccine effectiveness is calculated as 1-HR.

|  |  | |
| --- | --- | --- |
| Characteristic | Category | Adjusted HR (95%CI) |
| Vaccination status | Unvaccinated | - |
|  | One dose < 5 years ago | 0.041 (0.027-0.062) |
|  | One dose 5 to 9 years ago | 0.064 (0.035-0.118) |
|  | One dose 10 to 14 years ago | 0.084 (0.049-0.145) |
|  | One dose >= 15 years ago | 0.048 (0.027-0.088) |
|  | Two doses < 5 years ago | 0.002 (0.001-0.004) |
|  | Two doses 5 to 9 years ago | 0.004 (0.002-0.007) |
|  | Two doses 10 to 14 years ago | 0.012 (0.008-0.018) |
|  | Two doses >= 15 years ago | 0.003 (0.001-0.008) |
|  | Three doses < 5 years ago | 0.009 (0.002-0.035) |
|  | Three doses 5 to 9 years ago | 0.003 (0.000-0.024) |
| Age as at 31/12/2020 | 1-4 (2019-2016) | - |
| (Year of birth) | 5-11 (2015-2009) | 0.766 (0.332-1.770) |
|  | 12-16 (2008-2004) | 1.468 (1.084-1.988) |
|  | 17-18 (2003-2002) | 2.144 (1.487-3.092) |
|  | 19-21 (2001-1999) | 2.887 (2.084-4.000) |
|  | 22-30 (1998-1990) | 2.302 (1.648-3.216) |
| Gender | Male | - |
|  | Female | 0.999 (0.844-1.183) |
| Birth order | First born | - |
|  | Second born | 1.012 (0.774-1.323) |
|  | Third born | 1.164 (0.774-1.749) |
|  | Forth born | 1.287 (0.698-2.373) |
|  | Fifth or more | 1.444 (0.809-2.579) |
| Age first registered with | Born in Wales | - |
| Primary Care GP in Wales | Not born in Wales | 0.759 (0.623-0.924) |
| Broad ethnic group | White | - |
|  | Black/Asian/Mixed/Other | 0.471 (0.258-0.859) |
| Health board of residence | HB1 | - |
|  | HB2 | 4.448 (3.359-5.889) |
|  | HB3 | 1.623 (1.163-2.265) |
|  | HB4 | 0.517 (0.342-0.782) |
|  | HB5 | 0.257 (0.145-0.454) |
|  | HB6 | 3.728 (2.423-5.737) |
|  | HB7 | 0.590 (0.402-0.867) |
| Deprivation quintile | Most deprived | - |
| of residence | 2 | 1.066 (0.824-1.378) |
|  | 3 | 1.679 (1.303-2.165) |
|  | 4 | 1.193 (0.895-1.590) |
|  | Least deprived | 1.215 (0.916-1.612) |
| Ever eligible for free | No | - |
| school meals | Yes | 0.974 (0.804-1.181) |
|  | Pre-school age | 0.335 (0.102-1.101) |
| Total Primary Care | 0 | - |
| GP visits 01/01/2020 to | 1-4 | 1.421 (1.048-1.927) |
| 31/12/2020 | 5-9 | 1.291 (0.933-1.788) |
|  | 10+ | 1.394 (1.039-1.871) |
| Rural/Urban area of | Rural | - |
| residence | Urban | 1.622 (1.287-2.044) |

**Supplementary Table S11.** Estimated vaccine effectiveness for mumps containing vaccine against laboratory confirmed mumps infection by time since dose, in a retrospective cohort of 822 116 one to 30 year olds in Wales, UK. Vaccine effectiveness is calculated as 1-HR.

|  |  | |
| --- | --- | --- |
| Characteristic | Category | Adjusted HR (95%CI) |
| Vaccination status | Unvaccinated | - |
|  | One dose < 5 years ago | 0.168 (0.096-0.291) |
|  | One dose 5 to 9 years ago | 0.251 (0.130-0.483) |
|  | One dose 10 to 14 years ago | 0.235 (0.114-0.486) |
|  | One dose >= 15 years ago | 0.493 (0.344-0.706) |
|  | Two doses < 5 years ago | 0.064 (0.042-0.098) |
|  | Two doses 5 to 9 years ago | 0.137 (0.103-0.183) |
|  | Two doses 10 to 14 years ago | 0.357 (0.278-0.459) |
|  | Two doses >= 15 years ago | 0.501 (0.382-0.657) |
|  | Three doses < 5 years ago | 0.124 (0.054-0.283) |
|  | Three doses 5 to 9 years ago | 0.153 (0.079-0.296) |
|  | Three doses 10 to 14 years ago | 0.159 (0.079-0.318) |
|  | Three doses >= 15 years ago | 0.534 (0.321-0.887) |
| Age as at 31/12/2020 | 1-4 (2019-2016) | - |
| (Year of birth) | 5-11 (2015-2009) | 0.011 (0.000-86.201) |
|  | 12-16 (2008-2004) | 2.239 (1.345-3.726) |
|  | 17-18 (2003-2002) | 5.667 (3.365-9.543) |
|  | 19-21 (2001-1999) | 10.146 (6.171-16.683) |
|  | 22-30 (1998-1990) | 5.487 (3.318-9.073) |
| Gender | Male | - |
|  | Female | 0.728 (0.645-0.821) |
| Birth order | First born | - |
|  | Second born | 1.121 (0.976-1.288) |
|  | Third born | 1.138 (0.945-1.370) |
|  | Forth born | 0.930 (0.673-1.285) |
|  | Fifth or more | 0.919 (0.569-1.485) |
| Age first registered with | Born in Wales | - |
| Primary Care GP in Wales | Not born in Wales | 0.905 (0.797-1.026) |
| Broad ethnic group | White | - |
|  | Black/Asian/Mixed/Other | 0.911 (0.663-1.252) |
| Health board of residence | HB1 | - |
|  | HB2 | 1.959 (1.586-2.418) |
|  | HB3 | 1.718 (1.377-2.144) |
|  | HB4 | 1.437 (1.162-1.777) |
|  | HB5 | 1.868 (1.511-2.310) |
|  | HB6 | 0.912 (0.536-1.551) |
|  | HB7 | 1.214 (0.976-1.512) |
| Deprivation quintile | Most deprived | - |
| of residence | 2 | 1.068 (0.879-1.298) |
|  | 3 | 1.317 (1.082-1.602) |
|  | 4 | 1.325 (1.084-1.620) |
|  | Least deprived | 1.522 (1.260-1.839) |
| Ever eligible for free | No | - |
| school meals | Yes | 0.618 (0.531-0.719) |
|  | Pre-school age | 0.771 (0.448-1.325) |
| Total Primary Care | 0 | - |
| GP visits 01/01/2020 to | 1-4 | 0.978 (0.749-1.277) |
| 31/12/2020 | 5-9 | 1.830 (1.420-2.358) |
|  | 10+ | 2.092 (1.653-2.647) |
| Rural/Urban area of | Rural | - |
| residence | Urban | 0.906 (0.785-1.047) |

**Section 6.** Sensitivity analysis of Vaccine Effectiveness estimates for measles and mumps vaccination over time. Cox-regression analysis using an underlying calendar time scale.


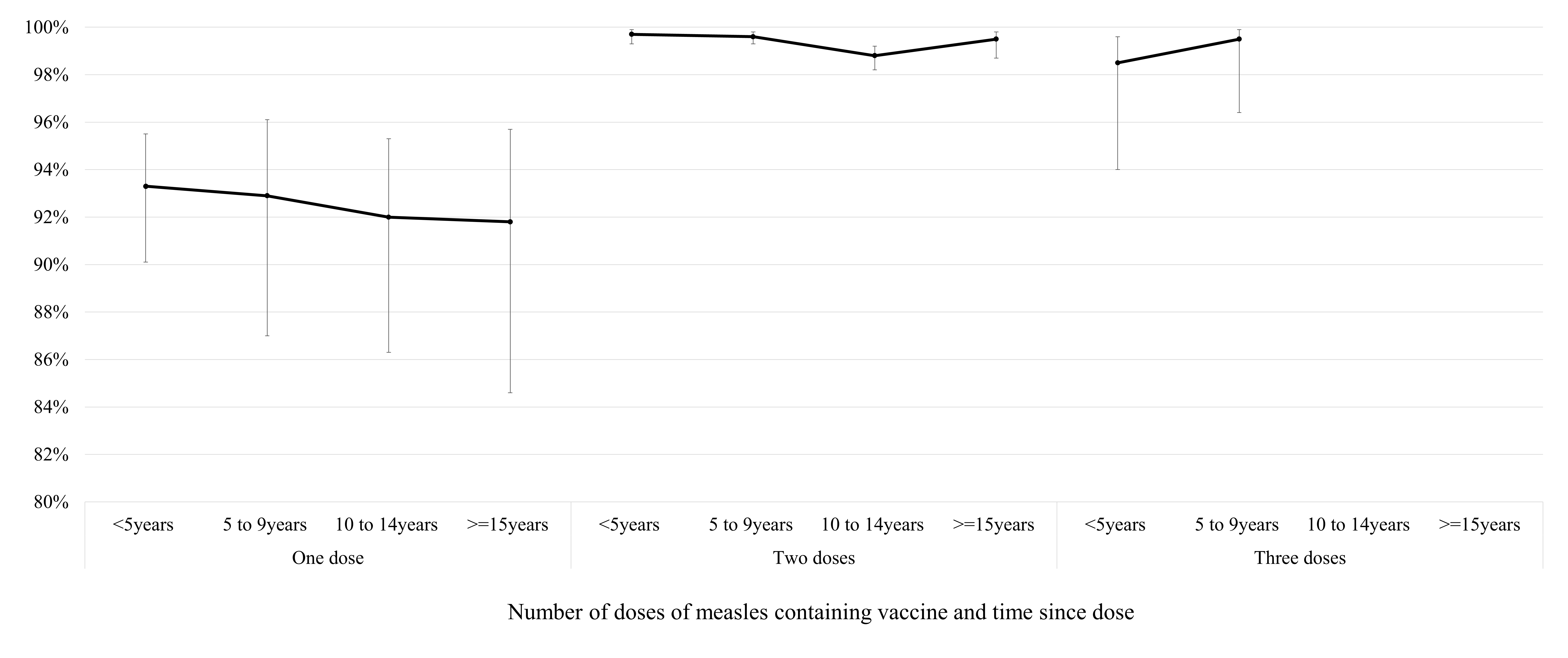
**Supplementary Figure S2.** Estimated vaccine effectiveness for measles containing vaccine against laboratory confirmed measles infection by time since dose, in a retrospective cohort of 822 116 one to 30 year olds in Wales, United Kingdom. ^a^ Results using an underlying calendar time scale.

^a^ Cox regression model adjusted for Age as at 31 December 2020, gender, birth order, age first registered with a Wales General Practice (GP), broad ethnic group, Health Board of residence, deprivation quintile of residence, eligibility for free school meals, total GP visits in the year preceding 31 December 2020 (or year before they exited the study), and residence in a rural/urban area.

**Supplementary Table S12.** Estimated vaccine effectiveness for measles containing vaccine against laboratory confirmed measles infection by time since dose, in a retrospective cohort of 822 116 one to 30 year olds in Wales, United Kingdom. ^a^ Results using an underlying calendar time scale.

|  |  | |
| --- | --- | --- |
| Characteristic | Category | Adjusted HR (95%CI) |
| Vaccination status | Unvaccinated | - |
|  | One dose < 5 years ago | 0.067 (0.045-0.099) |
|  | One dose 5 to 9 years ago | 0.071 (0.039-0.130) |
|  | One dose 10 to 14 years ago | 0.080 (0.047-0.137) |
|  | One dose >= 15 years ago | 0.082 (0.043-0.154) |
|  | Two doses < 5 years ago | 0.003 (0.001-0.007) |
|  | Two doses 5 to 9 years ago | 0.004 (0.002-0.007) |
|  | Two doses 10 to 14 years ago | 0.012 (0.008-0.018) |
|  | Two doses >= 15 years ago | 0.005 (0.002-0.013) |
|  | Three doses < 5 years ago | 0.015 (0.004-0.060) |
|  | Three doses 5 to 9 years ago | 0.005 (0.001-0.036) |

^a^ Cox regression model adjusted for Age as at 31 December 2020, gender, birth order, age first registered with a Wales General Practice (GP), broad ethnic group, Health Board of residence, deprivation quintile of residence, eligibility for free school meals, total GP visits in the year preceding 31 December 2020 (or year before they exited the study), and residence in a rural/urban area.

**Supplementary Figure S3.** Estimated vaccine effectiveness for mumps containing vaccine against laboratory confirmed mumps infection by time since dose, in a retrospective cohort of 822 116 one to 30 year olds in Wales, United Kingdom. ^a^ Results using an underlying calendar time scale.

^
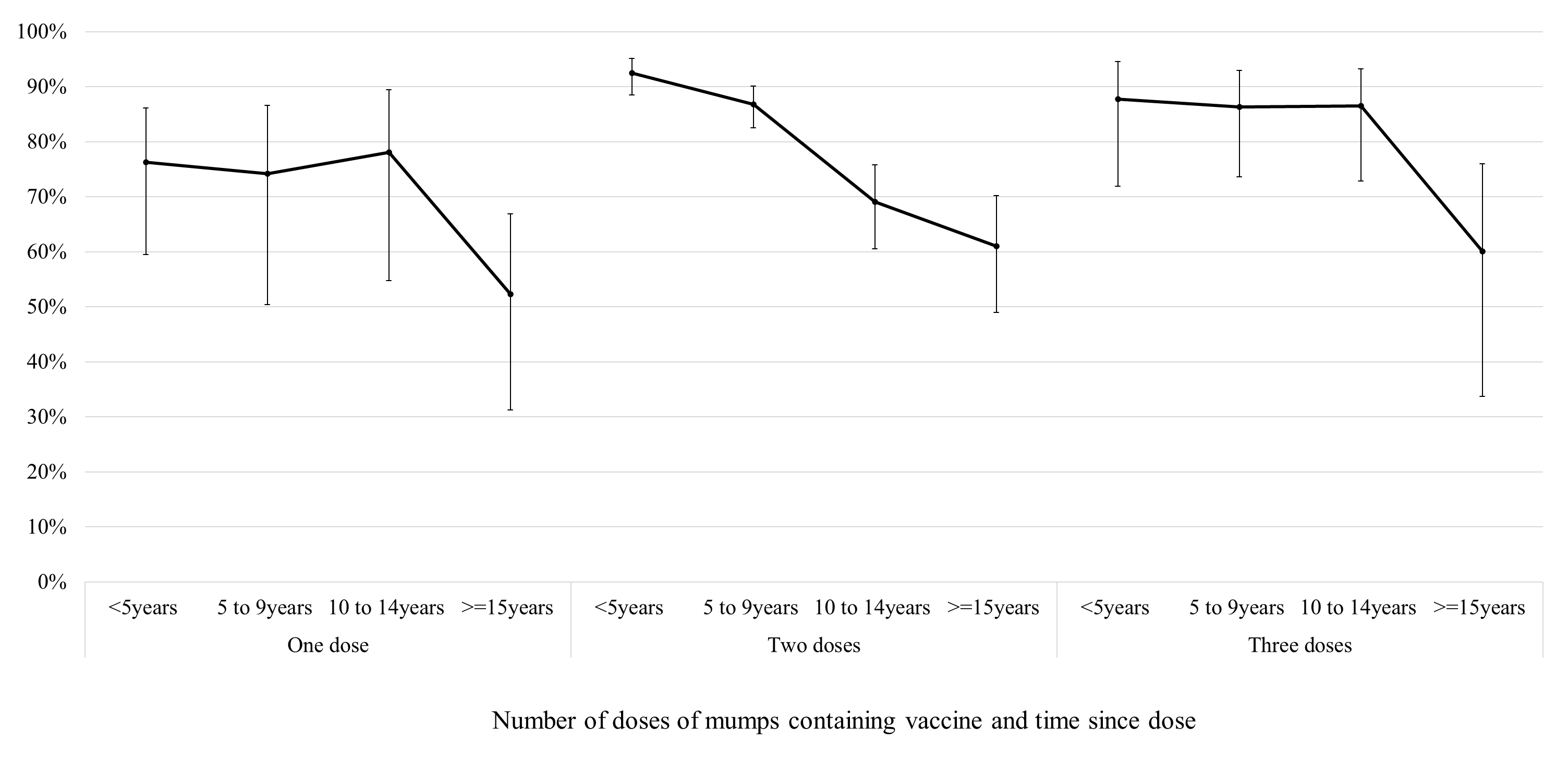
^

^a^ Cox regression model adjusted for Age as at 31 December 2020, gender, birth order, age first registered with a Wales General Practice (GP), broad ethnic group, Health Board of residence, deprivation quintile of residence, eligibility for free school meals, total GP visits in the year preceding 31 December 2020 (or year before they exited the study), and residence in a rural/urban area.

**Supplementary Table S13.** Estimated vaccine effectiveness for mumps containing vaccine against laboratory confirmed mumps infection by time since dose, in a retrospective cohort of 822 116 one to 30 year olds in Wales, United Kingdom. ^a^ Results using an underlying calendar time scale.

|  |  | |
| --- | --- | --- |
| Characteristic | Category | Adjusted HR (95%CI) |
| Vaccination status | Unvaccinated | - |
|  | One dose < 5 years ago | 0.237 (0.139-0.405) |
|  | One dose 5 to 9 years ago | 0.258 (0.134-0.496) |
|  | One dose 10 to 14 years ago | 0.219 (0.106-0.452) |
|  | One dose >= 15 years ago | 0.477 (0.331-0.687) |
|  | Two doses < 5 years ago | 0.075 (0.049-0.115) |
|  | Two doses 5 to 9 years ago | 0.132 (0.099-0.175) |
|  | Two doses 10 to 14 years ago | 0.309 (0.242-0.395) |
|  | Two doses >= 15 years ago | 0.390 (0.298-0.510) |
|  | Three doses < 5 years ago | 0.123 (0.054-0.281) |
|  | Three doses 5 to 9 years ago | 0.137 (0.071-0.264) |
|  | Three doses 10 to 14 years ago | 0.135 (0.068-0.271) |
|  | Three doses >= 15 years ago | 0.399 (0.240-0.663) |

^a^ Cox regression model adjusted for Age as at 31 December 2020, gender, birth order, age first registered with a Wales General Practice (GP), broad ethnic group, Health Board of residence, deprivation quintile of residence, eligibility for free school meals, total GP visits in the year preceding 31 December 2020 (or year before they exited the study), and residence in a rural/urban area.
